# Endometriosis and associations with risks of adverse pregnancy and perinatal outcomes: a case-control study in Egypt

**DOI:** 10.1186/s12884-025-07981-7

**Published:** 2025-09-01

**Authors:** Ahmed Elsayed Mansor, Mahmoud Kotb, Ola A. Harb, Walid S. H. Elsayed, Amany M. Abdallah, Mahmood Ahmed Osman, Ahmed Metwally Elkattawy

**Affiliations:** 1 Assistant professor of Gynecology and Obstetrics, Faculty of Medicine, Zagazig University, Zagazig, Egypt; 2 Assistant professor of Pathology, Faculty of Medicine, Zagazig University, Zagazig, Egypt; 3 Professor of Pathology, Faculty of Medicine, Tabuk University, Tabuk, Saudi Arabia; 4 Assistant professor of Family Medicine, Faculty of Medicine, Zagazig University, Zagazig, Egypt; 5 Charge Nurse, Zagazig University Hospitals, Zagazig University, Zagazig, Egypt; 6 Lecturer of Gynecology and Obstetrics, Faculty of Medicine, Zagazig University, Zagazig, Egypt

**Keywords:** Endometriosis, Surgical management, Placenta previa, Perinatal outcomes

## Abstract

**Background:**

Endometriosis and its association with adverse pregnancy and perinatal outcomes have recently drawn attention, pointing to increased risks of repeated caesarean sections, the occurrence of preterm births, and stillbirths.

**Patients and methods:**

This study included 25 pregnant women diagnosed with endometriosis and 25 pregnant women without endometriosis (control group). Maternal, fetal, and neonatal data were collected and compared between the endometriosis group and the control group concerning various maternal and neonatal parameters.

**Results:**

Patients with endometriosis were slightly older, primipara (*p* = 0.048), and conceived using assisted reproductive technology (< 0.001**) than the control group. There were significant differences in blood loss between the endometriosis and control groups (*p* = 0.01). There are significant differences between both groups regarding placenta previa and blood loss in either vaginal delivery or caesarean section, post-partum hemorrhage.

**Conclusions:**

Severe deep infiltrating pelvis or ovarian endometriosis is considered a risk factor for the occurrence of maternal complications such as placenta previa, as well as fetal and perinatal complications. Additionally, a past history of pre-pregnancy surgical management of endometriosis was associated with a high risk of the occurrence of placenta previa.

## Introduction

Endometriosis, which is defined as the presence of endometrial tissues outside the uterus might be presented with unspecific symptoms or might produce episodes of cyclic pain, dysmenorrhea, dyspareunia, and up to subfertility or infertility [1]. The rate of spontaneous pregnancy in female patients with mild to moderate endometriosis is about 75% of all affected women [2].

Associations between endometriosis (presence and severity) and adverse pregnancy and perinatal outcomes were recently taking attention pointing to increased risks or repeated caesarean sections, the occurrence of preterm births, and stillbirths [3].

But detailed evaluation of the association between endometriosis, preeclampsia, gestational diabetes, and intrauterine growth retardation was not assessed [4–6].

It is challenging to assess mechanisms, direct effects, and associations between endometriosis, pregnancy, and perinatal outcomes due to the limited number of small, single-centered studies on this topic, which have not provided sufficient data to be generalized [7]. Moreover, it is still uncertain whether surgical management or hormonal therapy of endometriosis before pregnancy affects pregnancy, maternal and perinatal outcomes [8].

Several large-scale studies and meta-analyses have highlighted the significant association between endometriosis and adverse pregnancy and perinatal outcomes. A meta-analysis involving nearly 700,000 women reported that endometriosis is linked to an increased risk of miscarriage in spontaneous conceptions, as well as higher odds of antepartum hemorrhage, postpartum hemorrhage, preterm birth, stillbirth, and placenta previa. Similarly, another systematic review found that women with endometriosis who conceived through assisted reproductive technologies (ART) had a notably elevated risk of placenta previa, with odds ratios exceeding 5.5. Furthermore, a population-based cohort study of over 900,000 births demonstrated increased risks of preeclampsia, placenta previa, and preterm birth among women with endometriosis, regardless of conception method [9–11].

Furthermore, recent meta-analyses report that endometriosis affects approximately 18% of women, with even higher prevalence among those with infertility or chronic pelvic pain, underscoring the widespread burden of this condition and the importance of effective pre-pregnancy identification and management strategies [12].

Therefore, the present study aimed to assess the effect of endometriosis on the occurrence of adverse pregnancy, and perinatal outcomes and to clarify whether pre-pregnancy surgical management of endometriosis affects these outcomes.

## Patients and methods

A retrospective case-control study was conducted at the Department of Obstetrics and Gynecology, Faculty of Medicine, Zagazig University Hospitals, over six years from January 2017 to December 2022 to compare maternal and neonatal outcomes among pregnant women with and without endometriosis. The study was approved by the Institutional Review Board (IRB) of the Faculty of Medicine, Zagazig University (ZU-IRB# 656/22-Dec-2024).

### Inclusion and exclusion criteria

The inclusion criteria comprised pregnant women diagnosed with ovarian endometriomas either during the first trimester of pregnancy or before pregnancy in the endometriosis group. Patients with endometriosis were further categorized into two subgroups:

1. The surgical treatment subgroup included women with a history of surgical management (e.g., ovarian cystectomy, excision of endometriotic implants, and adhesiolysis).
2. The medical treatment subgroup consisted of women who received only medical management or were conservatively monitored without surgical intervention.

The exclusion criteria were:

- Gestational age is less than 23 weeks at the time of birth.
- Patients with fetal abnormalities.
- Multiple gestations.
- Incomplete or missing medical records.

Following the application of inclusion and exclusion criteria, a total of 50 pregnant women were included in the study: 25 in the endometriosis group and 25 in the control group (women without endometriosis).

### Operational definitions

#### Endometriosis

A gynecological disorder marked by the presence of glandular and stromal tissue resembling the endometrium at ectopic sites outside the uterine cavity. The condition is driven by complex mechanisms, prominently involving hormonal imbalances, especially elevated estrogen levels which play a central role in its initiation, progression, and persistence [13].

#### Placenta previa

A pregnancy complication characterized by the partial or complete implantation of the placenta over the internal cervical os. It typically occurs during the second or third trimester, though it may also be detected earlier in pregnancy. Placenta previa is a leading cause of antepartum hemorrhage and may result in significant maternal and fetal complications due to vaginal bleeding. Its incidence ranges from approximately 0.28–2%, affecting about 3 to 5 per 1,000 pregnancies [14].

#### Preterm birth

The delivery of a live infant before 37 completed weeks of gestation. It affects approximately 11% of births globally and represents a major contributor to neonatal morbidity, mortality, and long-term health complications [15, 16].

#### Postpartum hemorrhage (PPH)

A blood loss of 500 ml or more following childbirth. It is a leading cause of maternal morbidity and mortality globally, accounting for approximately 27% of maternal deaths [17].

Fetal Growth Restriction (FGR), also known as Intrauterine Growth Restriction (IUGR), is defined as a condition in which the fetus does not reach its genetically predetermined growth potential. It is typically identified when the fetal growth rate is below what is expected for its gestational age, sex, and race. Although often used interchangeably with Small for Gestational Age (SGA), FGR specifically reflects a pathological restriction in growth. It may be diagnosed when the estimated fetal weight or birth weight falls below the 10th percentile for gestational age and is associated with an increased risk of fetal and neonatal morbidity and mortality [18].

### Data collection

All maternal, fetal, and neonatal data were extracted from patient medical records. The diagnosis of endometriosis was confirmed through laparoscopic evaluation followed by histopathological confirmation.

Maternal baseline characteristics assessed included age, parity, pre-pregnancy body mass index (BMI), presence of pre-pregnancy medical conditions such as hypertension and diabetes mellitus, and the use of assisted reproductive techniques (ART).

Maternal outcome parameters evaluated were gestational age, mode of delivery, estimated blood loss during delivery, and degree and types of complications such as gestational diabetes mellitus (GDM), preterm birth (defined as delivery before 37 weeks of gestation), postpartum hemorrhage, and placenta previa.

Neonatal outcome parameters assessed included birth length, birth weight, Apgar scores at one and five minutes, umbilical artery pH, and the need for admission to the neonatal intensive care unit (NICU).

Comparative analyses were performed between endometriosis and control groups concerning maternal and neonatal outcome parameters.

### Statistical analysis

All statistical analyses were performed using R software version 3.5.0 (www.r-project.org) and EZR (Saitama Medical Center, Jichi Medical University, Saitama, Japan). Maternal and neonatal parameters were compared using the chi-squared test for categorical variables, and either the unpaired t-test or Mann–Whitney U test for continuous variables, as appropriate. Crude odds ratios (CORs) were calculated using bivariate logistic regression, and adjusted odds ratios (aORs) with 95% confidence intervals (CIs) were estimated using multivariate logistic regression after controlling for potential confounders, including pre-pregnancy body mass index (BMI) and maternal age at delivery. A p-value of less than 0.05 was considered statistically significant.

## Results

Patients with endometriosis were slightly older (*p* = 0.03) and more likely to be primipara (*p* = 0.048) and conceive using ART (< 0.001**) than the control group. There is a statistically significant difference between the studied groups regarding parity (64% versus 36% within the case and control group were primipara) and ART (36% versus 12% within the case and control group received ART). There is a statistically non-significant difference between the studied groups regarding age, BMI, pre-pregnancy diabetes, or hypertension (Table 1).

**Table 1:** Comparison between the studied groups regarding baseline data

	Endometriosis group *N* = 25(%)	Control group *N* = 25 (%)	χ^2^	*p*
Age [mean ± SD]	30.56 ± 6.31	29.96 ± 3.96	0.818^¥^	0.414
BMI [mean ± SD]	24.37 ± 3.39	24.58 ± 4.16	−0.401^¥^	0.689
Primipara	16 (64%)	9 (36%)	3.92	0.048*
Pre-pregnancy DM HTN	5 (5%) 7 (7%)	3 (3%) 6 (6%)	Fisher 0.082	0.721 0.774
ART	36 (36%)	12 (12%)	15.789	< 0.001**

*p < 0.05 is statistically significant ^¥^independent sample t

There were significant differences in blood loss between the endometriosis and control groups (*p* = 0.01). There were no significant differences in gestational age, delivery mode, blood loss on instrumental delivery, and emergency cesarean section between the two groups.

### Regarding pregnancy complications

There is a statistically significant difference between the studied groups regarding placenta previa (36% versus 7% within the case and control group had placenta previa) and blood loss in either vaginal delivery and CS (both were significantly higher among the case group), post-partum hemorrhage (36% versus 16% within the case and control group had postpartum hemorrhage). There is a statistically non-significant difference between the studied groups regarding mode of delivery, placenta abruption, GDM, or hypertension (Table 2).

**Table 2:** Comparison between the studied groups regarding maternal outcome

	Endometriosis group*N* = 25(%)	Control group*N* = 25 (%)	χ^2^	*p*
Mode of delivery				
Vaginal delivery	43 (43%)	55 (55%)	2.881	0.09
CS	57 (57%)	45 (45%)		
Blood loss	Median (IQR)	Median (IQR)	Z	P
Vaginal delivery CS	500(300–857.5) 980(725–1225)	210(150–310) 690(555–780)	−5.448 −5.6	< 0.001** < 0.001**
GDM	7 (4%)	3 (3%)	1.684	0.194
Hypertension	9 (9%)	5(5%)	1.229	0.268
Placenta previa	36 (36%)	7 (7%)	24.915	< 0.001**
Placental abruption	3 (3%)	0 (0%)	Fisher	0.246
Post partum hemorrhage	36 (36%)	16 (16%)	10.395	0.001**

*p < 0.05 is statistically significant, ^¥^independent sample t test, χ^2^chi square test, Z Mann Whitney test

We performed a normality test for continuous variables.

Neonatal outcomes such as birth weight, height, SGA, and NICU admission rates were similar between the two groups. There is a statistically non-significant difference between the studied groups regarding neonatal outcomes (Table 3).

**Table 3:** Comparison between the studied groups regarding neonatal outcome

	Endometriosis group *N* = 100(%)	Control group *N* = 100(%)	χ^2^	*p*
GA[mean ± SD] < 37 week (preterm)	37.88 ± 1.81 16 (16%)	37.96 ± 1.27 11 (11%)	−0.367^¥^ 1.07	0.714 0.301
Male gender	75 (76%)	72 (72%)	0.231	0.631
Height [mean ± SD]	24.37 ± 3.39	24.58 ± 4.16	−0.949^¥^	0.344
APGAR < 7 At 1 min At 5 min	8 (8%) 4 (4%)	4 (4%) 3 (3%)	1.418 Fisher	0.234 > 0.999
Umbilical PH < 7	4 (4%)	0 (0%)	Fisher	0.121
SGA	7 (7%)	3 (3%)	1.684	0.194
LBW	11 (11%)	5 (5%)	2.446	0.118
NICU admission	4(16%)	2(8%)	Fisher	0.667

*p < 0.05 is statistically significant ^¥^independent sample t test χ^2^chi square test Z Mann Whitney test

Univariate and multivariate analyses were performed to identify the independent risk factors for placenta previa. In univariate analysis, endometriosis, age ≥ 35 years, and ART significantly increase the risk of placenta previa by 6.47, 10.5, and 11.9 folds, respectively. BMI ≥ 25 kg/m^2^ and multipara non-significantly increase risk by 3.13 and 3.45 folds, respectively.

In multivariate analysis, endometriosis and multipara significantly independently increase risk by 14.2 and 9.83 folds respectively. ART, BMI ≥ 25 kg/m^2,^ and age ≥ 35 years non-significantly increase risk by 4.93, 3.01, and 3.67 folds respectively. The multivariate analysis was adjusted for endometriosis, pre-pregnancy BMI ≥ 25 kg/m2, maternal age ≥ 35 years old, ART, and multipara (Table 4). Endometriosis was identified as an independent risk factor for placenta previa (aOR, 3.19; 95% CI, 1.56–6.50, *p* < 0.01).

**Table 4:** Univariate and multivariate analysis of risk factors for placenta previa among studied patients

Predictors	COR (95% CI)	*p*	AOR (95% CI)	*p*
Endometriosis	6.47(1.23–34.1)	< 0.001**	14.2(1.24–162.9)	< 0.001**
BMI ≥ 25 kg/m^2^	3.13(0.78–12.57)	0.001**	3.01(0.41–22.21)	0.03*
Age (≥ 35 years)	10.5(2.18–50.69)	< 0.001**	3.67(0.47–28.68)	0.013*
ART	11.9(2.54–55.85)	< 0.001**	4.93(0.72–33.86)	0.001**
Multipara	3.45(0.79–15.01)	0.001**	9.83(1.02–94.66)	< 0.001**

*COR* Crude odds ratio, *AOR* Adjusted odds ratio, *CI* Confidence interval

*p<0.05 is statistically significant

In univariate analysis, age ≥ 35 years and ART significantly increase the risk of placenta previa by 7.07 and 4.43 folds respectively. Endometriosis, BMI ≥ 25 kg/m^2,^ and multipara non-significantly increase risk by 2.95, 2.95, and 1.88 folds respectively. In multivariate analysis, Endometriosis, multipara ART, BMI ≥ 25 kg/m^2^, age ≥ 35 years, and ART non-significantly increase risk by 3.02, 2.04, 2.25, 3.23, and 1.78 folds respectively (Table 5).

**Table 5:** Univariate and multivariate analysis of risk factors for post-partum hemorrhage among studied patients

Predictors	COR (95% CI)	*P*	AOR (95% CI)	*p*
Endometriosis	2.95(0.77–11.34)	0.002*	3.02(0.55–16.57)	0.011*
BMI ≥ 25 kg/m ^2^	2.95(0.8–10.9)	0.001**	2.25(0.5–10.03)	0.034*
Age (≥ 35 years)	7.07(1.57–31.86)	< 0.001**	3.23(0.57–16.57)	0.008*
ART	4.43(1.1–17.92)	< 0.001**	1.78(0.32–9.74)	0.186
Multipara	1.88(0.52–6.85)	0.055	2.04(0.5–10.03)	0.082

*COR* Crude odds ratio *AOR* Adjusted odds ratio *CI* Confidence interval

*p<0.05 is statistically significant

## Discussion

In the present study, we assessed associations between endometriosis, maternal and perinatal neonatal outcomes and we showed that endometriosis was an important risk factor for the occurrence of placenta previa, which is consistent with the results reported by Miura et al. (2019) [8].

Moreover, we showed that females complaining of endometriosis with a past history of surgical management before pregnancy have high risks of placenta previa and patients who received only hormone therapies or patients diagnosed with ovarian endometriosis accidentally in the first trimester of pregnancy have no increased risk.

Our findings are consistent with previous studies that have demonstrated an association between endometriosis and the occurrence of placenta previa [8, 19–21]. We showed that endometrosis was associated with preterm delivery, hypertensive disorders of pregnancy, small gestational age, and postpartum hemorrhage.

Our findings differ from those of previous studies [22, 23], which reported that endometriosis does not increase maternal or perinatal risks. These discrepancies may be attributed to variations in sample size, studies cohort, and research designs, as noted in studies by Berlac et al. (2017), Shmueli et al. (2019), and Harada et al. (2016) [24–26].

We showed the association between endometriosis and intrauterine fetal growth restriction as we showed that fetal birth weights are lower than two standard deviations from the mean weight for the same gestational age or less than the 10th percentile of the normal weight for the same age.

Pathogenesis of the association between endometriosis and pregnancy complications might be explained by many theories; chronic inflammation and inflammation-associated chemical mediators as; cyclooxygenase-2, prostaglandin E2, and interleukin-8 [27]. Additionally, it was found that progesterone-resistant endometrium, vascularized environment and adhesions that occurred due to endometriosis might be responsible for many pregnancy-associated complications [27–29].

The increased incidence of placenta previa in patients with endometriosis is mostly due to uterine hyperperistalsis which might lead to abnormal implantation of the blastocyst [30, 31], additionally, the presence of dense adhesions in the pelvis leads to inhibition of placenta migration away from the uterine internal ostium.

It was previously demonstrated that placenta previa was more observed in patients with recto-vaginal endometriosis than in patients with ovarian or peritoneal lesions [31]. Moreover, severe cases of deep infiltrating endometriosis (DIE) and stage IV endometriosis were associated with a higher placenta previa incidence in subsequent pregnancies [32, 33].

Regarding associations between pre-pregnancy surgical excision of endometriosis and increased risks of placenta previa, women with a history of surgical treatment for endometriosis prior to pregnancy demonstrated a notably higher incidence of placenta previa. This is evident from our results showing a significantly increased rate of placenta previa in the endometriosis group (36%) compared to the control group (7%). Our multivariate analysis also identified endometriosis as an independent risk factor for placenta previa (aOR: 3.19, 95% CI: 1.56–6.50, *p* < 0.01). Thus, the increased risks of placenta previa in patients with severe endometriosis who underwent surgical management before pre-pregnancy are caused by severe stage of endometriosis before surgery, endometriosis recurrence, or due to adhesions caused by operative intervention [24, 32].

### Points of strength

Most studies tried to assess the effect and severity of endometriosis on fertility. But we noticed that deep infiltrating pelvis is a risk factor for the occurrence of maternal complications such as placenta previa, fetal and perinatal complications. Our study highlighted these finding by this comparative study to prove the current knowledge in such topic, particularly after the high incidence and complicated management of deep pelvic endometriosis.

### Limitations of our study

First, in the nonsurgical treatment group, we depend on ultrasound, MRI, and the presence of symptoms in the diagnosis of endometriosis which are less accurate diagnostic methods than laparoscopy which is the gold standard.

Second, the study is a retrospective and single-center study that included a relatively small number of patients.

## Conclusions

Endometriosis, particularly in women with a history of pre-pregnancy surgical treatment, is associated with increased risks of placenta previa and postpartum hemorrhage. These complications emphasize the need for early identification and tailored antenatal management in this population. Additionally, women who received only medical or hormonal therapies did not demonstrate increased maternal or neonatal risks. This highlights the differential impact of disease severity and treatment approach on pregnancy outcomes.

## Recommendations

Large-scale, prospective, and multicenter studies are recommended to investigate associations between endometriosis site, severity, surgical treatment, and risks of placenta previa in a subsequent pregnancy in addition to associated with fetal and perinatal outcomes.

Conduct educational sessions for obstetricians, midwives, and residents to increase awareness about endometriosis-related pregnancy risks and proper clinical management.

## Data Availability

The dataset generated and/or analyzed during the current study is available from the corresponding author upon reasonable request.
